# Analysis of the susceptibility of refractory hepatitis C virus resistant to nonstructural 5A inhibitors

**DOI:** 10.1038/s41598-024-67169-5

**Published:** 2024-07-16

**Authors:** Ai Toyodome, Seiichi Mawatari, Hiromi Eguchi, Midori Takeda, Kotaro Kumagai, Ohki Taniyama, Sho Ijuin, Haruka Sakae, Kazuaki Tabu, Kohei Oda, Masanori Ikeda, Akio Ido

**Affiliations:** 1 Digestive and Lifestyle Diseases, Department of Human and Environmental Sciences, Kagoshima University Graduate School of Medical and Dental Sciences, 8‑35‑1 Sakuragaoka, Kagoshima, 890‑8544 Japan; 2 Division of Biological Information Technology, Joint Research Center for Human Retrovirus Infection, Kagoshima University, 8‑35‑1 Sakuragaoka, Kagoshima, 890‑8544 Japan

**Keywords:** Viral hepatitis, Viral infection

## Abstract

Resistance-associated substitutions (RASs) of hepatitis C virus (HCV) affect the efficacy of direct-acting antivirals (DAAs). In this study, we aimed to clarify the susceptibility of the coexistence of nonstructural (NS) 5A Q24K/L28M/R30Q (or R30E)/A92K RASs, which were observed in patients with DAAs re-treatment failure and to consider new therapeutic agents. We used a subgenomic replicon system in which HCV genotype 1B strain 1B-4 was electroporated into OR6c cells derived from HuH-7 cells (Wild-type [WT]). We converted WT genes to NS5A Q24K/L28M/R30Q/A92K or Q24/L28K/R30E/A92K. Compared with the WT, the Q24K/L28M/R30Q/A92K RASs was 36,000-fold resistant to daclatasvir, 440,000-fold resistant to ledipasvir, 6300-fold resistant to velpatasvir, 3100-fold resistant to elbasvir, and 1.8-fold resistant to pibrentasvir. Compared with the WT, the Q24K/L28M/R30E/A92K RASs was 640,000-fold resistant to daclatasvir and ledipasvir, 150,000-fold resistant to velpatasvir, 44,000-fold resistant to elbasvir, and 1500-fold resistant to pibrentasvir. The Q24K/L28M/R30E/A92K RASs was 816.3 times more resistant to pibrentasvir than the Q24K/L28M/R30Q/A92K RASs. Furthermore, a combination of pibrentasvir and sofosbuvir showed therapeutic efficacy against these RASs. Combination regimens may eradicate HCV with NS5A Q24K/L28M/R30E/A92K RASs.

## Introduction

Hepatitis C virus (HCV) is a ribonucleic acid (RNA) virus belonging to the family *Flaviviridae* that was identified in 1989 by Choo et al.^1^. Approximately 70% of patients infected with HCV develop chronic hepatitis. Persistent liver inflammation leads to liver fibrosis, which progresses to cirrhosis and hepatocellular carcinoma (HCC)^2^.

In 2014, the first interferon-free direct-acting antivirals (DAAs), daclatasvir (a nonstructural (NS) 5A inhibitor) and asunaprevir were authorized for the treatment of chronic hepatitis or compensated cirrhosis in patients with HCV genotype 1^3^. Subsequently, various regimens have been approved^4–9^. Currently, DAA therapy is available for chronic hepatitis C for as little as 8 weeks, and regimens for re-treatment and non-compensated cirrhosis have also been established, resulting in treatment with minimal side effects and a high sustained virological response (SVR) rate of > 95%.

The results of a domestic phase III trial showed that the SVR rate of daclatasvir and asunaprevir combination therapy differed significantly between 91.3 and 43.3%, depending on the baseline resistance-associated substitutions (RASs) of Y93H in the NS5A region^3^. Therefore, at our hospital and affiliated facilities (Kagoshima Liver Study Group), we confirmed RASs in all patients before DAAs administration, and treatment selection was based on the obtained results^10–12^. However, despite the administration of daclatasvir and asunaprevir to patients without baseline NS5A Y93H RAS, approximately 18% of patients did not achieve SVR. The presence of baseline NS5A Q24/L28 and/or R30 RASs was associated with virologic failure of daclatasvir and asunaprevir therapy. Therefore, we concluded that the coexistence of baseline RASs other than NS5A L31 and Y93 may affect the therapeutic effectiveness of daclatasvir and asunaprevir therapy^10^.

According to the Hepatitis C Treatment Guidelines in Japan, glecaprevir/pibrentasvir or sofosbuvir/velpatasvir and ribavirin are indicated as regimens for re-treatment of genotypes 1 and 2 DAA in patients with pre-treatment failure^13^. In a nationwide multicenter study in Japan that analyzed cases of DAA treatment failure, P32del or A92K RAS in NS5A conferred strong resistance to pan-genotypic NS5A inhibitors. P32del in NS5A was found in 4.0% of cases and A92K in 2.4%^14^. In our study group, 48 patients were re-treated with glecaprevir/pibrentasvir, and patients without RAS, with one RAS, or with Y93H RAS achieved SVR; however, two patients with the coexistence of NS5A Q24, L28, R30, and A92K RASs and one patient with a P32 deletion RAS did not achieve SVR. A comparison of pre- and post-treatment RAS in patients with NS5A Q24/L28/R30/A92K RASs showed that R30Q changed to R30E after virologic failure^12^. The coexistence of NS5A Q24K/L28M/R30E/A92K RASs may make the virus highly resistant to glecaprevir/pibrentasvir. However, there have been no in vitro studies on NS5A Q24K/L28M/R30E/A92K RASs. We aimed to clarify the susceptibility of the coexistence of NS5A Q24K/L28M/R30Q (or R30E)/A92K RASs, which were observed in patients with treatment failure, and to explore new therapeutic agents.

## Results

### Frequency of NS5A RASs before and after DAA treatment in glecaprevir/pibrentasvir failure

We analyzed the amino acid substitutions in the NS5A regions of two patients with glecaprevir/pibrentasvir failure before and after treatment using direct sequencing and ultra-deep sequencing. Table 1 shows the results of ultra-deep sequencing, highlighting the evolution of NS5A RASs over time. In Case 1, most amino acid substitutions were Q24K/L28M/R30Q/A92T or A before daclatasvir and asunaprevir therapy. After the failure of daclatasvir and asunaprevir treatment (prior to glecaprevir/pibrentasvir treatment), R30E or A92K were detected, which were not present before treatment and were caused by single nucleotide substitutions. After glecaprevir/pibrentasvir failure, most amino acid substitutions changed to Q24K/L28M/R30E/A92K.

**Table 1:** Frequency of NS5A RASs before and after treatment in patients with G/P failure.

Reference	Amino acid, frequency (%)																			
	Q24		L28		R30		L31		P32		F37		Q54		P58		A92		Y93	
Case 1																				
Before DCVASV	K	99.6	M	99.6	Q	99.6	L	99.9	P	99.8	F	99.6	Q	99.6	P	99.6	T	97	Y	98.9
	Q	0.2	L	0.2	R	0.2					V	0.2	H	0.3			A	2.9	N	0.6
																			H	0.3
Before G/P	K	99.7	M	99.3	Q	39.1	L	72.6	P	99.9	F	98.9	Q	99.9	P	99.8	K	85.5	Y	99.7
			V	0.6	E	60.8	V	27.5			L	1.0					T	8.4		
																	E	4.6		
																	R	1.0		
After G/P failure	K	99.9	M	99.9	E	99.9	L	99.9	P	99.9	F	99.9	Q	99.9	P	99.9	K	99.9	Y	99.9
Case 2																				
Before G/P	K	99.2	M	99.4	Q	97.5	L	99.9	P	99.9	L	99.9	Q	99.9	P	99.6	T	94.5	Y	99.8
	Q	0.5	V	0.5	E	2.5											K	3.0		
	E	0.3																		
After G/P failure	K	99.9	M	99.9	E	99.8	L	99.9	P	99.9	L	99.9	Q	99.9	P	99.9	K	99.9	Y	99.9

All amino acid frequencies were detected by ultra-deep sequencing.

HCV-Con1 (accession no. AJ238799) or HCV-J (accession no. D90208) were used as references.

*DCVASV* daclatasvir and asunaprevir, *G/P* glecaprevir/pibrentasvir, *aa* amino acid, *RAS* resistance-associated substitution.

In Case 2, most amino acid substitutions were Q24K/L28M/R30Q/A92T, with 2.5% R30E and 3.0% A92K detected after daclatasvir and asunaprevir failure (prior to glecaprevir/pibrentasvir treatment). After glecaprevir/pibrentasvir failure, most amino acid substitutions changed to Q24K/L28M/R30E/A92K.

### Susceptibility of each cell to NS5A inhibitor

**Figure 1:**
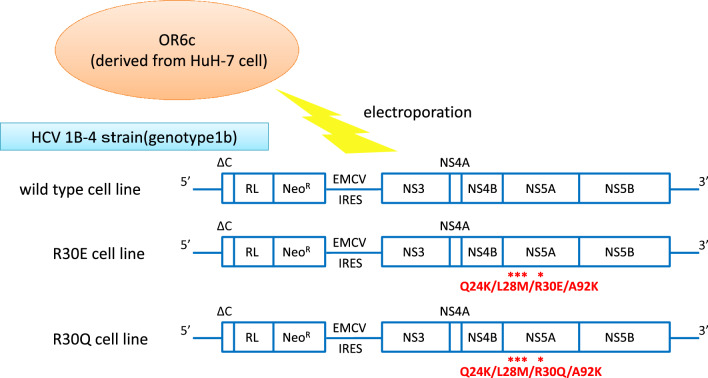
Schematic representation of subgenomic replicon and their derivatives with NS5A resistance-associated substitutions. OR6c cells derived from HuH-7 cells and the 1B-4 strain of HCV genotype 1B were used as subgenomic replicons. *RL* *Renilla* luciferase, *Neo*^*R*^ neomycin phosphotransferase, *EMCV* encephalomyocarditis virus, *IRES* internal ribosome entry site, *NS* nonstructural, *HCV* hepatitis C virus.

**Figure 2:**
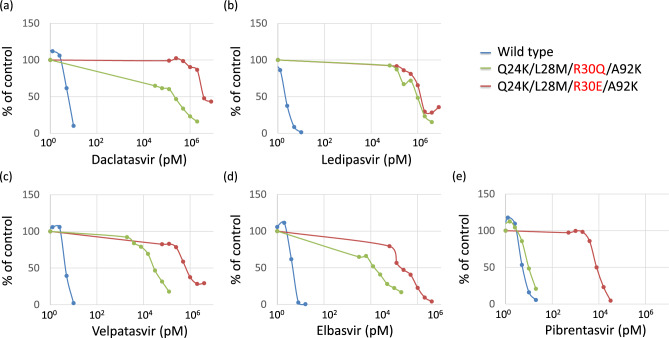
Susceptibility of HCV 1B-4 strain and its derivatives with NS5A RASs to NS5A inhibitors. We seeded 1.5 × 10^4^ cells/well in a 24-well plate; after 24 h of culture, anti-HCV agents were added and treated for 72 h. Susceptibility to NS5A inhibitors: (**a**) daclatasvir, (**b**) ledipasvir, (**c**) velpatasvir, (**d**) elbasvir, and (**e**) pibrentasvir was analyzed using the *Renilla* luciferase assay. *NS* nonstructural, *HCV* hepatitis C virus, *RAS* resistance-associated substitution.

To assess the susceptibility of NS5A Q24K/L28M/R30E/A92K RASs, we used a subgenomic replicon system in which HCV genotype 1B strain 1B-4 was electroporated into OR6c cells derived from HuH-7 cells. We designated these cells as Wild-type (WT). We converted WT genes to NS5A Q24K/L28M/R30Q/A92K or Q24K/L28M/R30E/A92K (Fig. 1). The susceptibilities of the WT, Q24K/L28M/R30Q/A92K, and Q24K/L28M/R30E/A92K to various NS5A inhibitors were examined. Susceptibility to the NS5A inhibitors (daclatasvir, ledipasvir, velpatasvir, elbasvir, and pibrentasvir) was analyzed using the *Renilla* Luciferase assay, as shown in Fig. 2 and Table 2. The effective concentration required for 50% inhibition (EC50) was calculated for the WT, Q24K/L28M/R30Q/A92K, and Q24K/L28M/R30E/A92K. The EC50 values for WT, Q24K/L28M/R30Q/A92K, and Q24K/L28M/R30E/A92K were 6.1 pM, 220 nM, and 3.9 μM for daclatasvir (Fig. 2a, Table 2); 2.2 pM, 960 nM, and 1.4 μM for ledipasvir (Fig. 2b, Table 2); 4.6 pM, 29 nM, and 710 nM for velpatasvir (Fig. 2c, Table 1); 4.8 pM, 15 nM, and 210 nM for elbasvir (Fig. 2d, Table 2); and 5.5 pM, 9.8 pM, and 8.0 nM for pibrentasvir (Fig. 2e, Table 2), respectively. Compared with the WT, the Q24K/L28M/R30Q/A92K was 36,000-fold resistant to daclatasvir, 440,000-fold resistant to ledipasvir, 6300-fold resistant to velpatasvir, 3100-fold resistant to elbasvir, and 1.8-fold resistant to pibrentasvir (Table 2). Compared with the WT, the Q24K/L28M/R30E/A92K was 640,000-fold resistant to daclatasvir, 640,000-fold resistant to ledipasvir, 150,000-fold resistant to velpatasvir, 44,000-fold resistant to elbasvir, and 1500-fold resistant to pibrentasvir (Table 2). Compared with the Q24K/L28M/R30Q/A92K, the Q24K/L28M/R30E/A92K was 17.7-fold resistant to daclatasvir, 1.5-fold resistant to ledipasvir, 24.5-fold resistant to velpatasvir, 14-fold resistant to elbasvir, and 816.3-fold resistant to pibrentasvir (Table 2).

**Table 2:** Susceptibility of NS5A RAS to NS5A inhibitors.

Cell line (OR6c/1B-4)	Daclatasvir		Ledipasvir		Velpatasvir		Elbasvir		Pibrentasvir	
	EC50 (pM)	Fold resistance	EC50 (pM)	Fold resistance	EC50 (pM)	Fold resistance	EC50 (pM)	Fold resistance	EC50 (pM)	Fold resistance
WT	6.1	1.0	2.2	1.0	4.6	1.0	4.8	1.0	5.5	1.0
Q24K/L28M/R30Q/A92K	2.2 × 10^5^	3.6 × 10^4^	9.6 × 10^5^	4.4 × 10^5^	2.9 × 10^4^	6.3 × 10^3^	1.5 × 10^4^	3.1 × 10^3^	9.8	1.8
Q24K/L28M/R30E/A92K	3.9 × 10^6^	6.4 × 10^5^	1.4 × 10^6^	6.4 × 10^5^	7.1 × 10^5^	1.5 × 10^5^	2.1 × 10^5^	4.4 × 10^4^	8.0 × 10^3^	1.5 × 10^3^
Comparison of R30Q and R30E cell line	17.7-fold		1.5-fold		24.5-fold		14-fold		816.3-fold	

The fold change in resistance was compared with that of the WT.

*EC50* effective concentration required to inhibit 50%, *WT* wild-type, *NS* nonstructural, *RAS* resistance-associated substitution.

### Susceptibility of cells to NS5B inhibitors and agents with anti-HCV effects other than DAAs

Regarding the NS5B inhibitor sofosbuvir, the EC50 values of WT, Q24K/L28M/R30Q/A92K, and Q24K/L28M/R30E/A92K were 113.3 nM, 79.9 nM, and 100.5 nM, respectively (Table 3). No resistance to NS5B inhibitors was observed in the Q24K/L28M/R30Q/A92K and Q24K/L28M/R30E/A92K RASs compared with in the WT. Regarding agents with anti-HCV effects other than DAAs, the EC50 values for WT, Q24K/L28M/R30Q/A92K, and Q24K/L28M/R30E/A92K were 48.3 ng/L, 103 ng/L, and 96.2 ng/L for interferon-alpha; 1.5 µM, 1.7 µM, and 2.7 µM for fluvastatin; and 0.9 ng/mL, 1.5 ng/mL, and 3.5 ng/mL for oncostatin M, respectively (Table 3). The Q24K/L28M/R30Q/A92K and Q24K/L28M/R30E/A92K RASs were mildly resistant to interferon-alpha, fluvastatin, and oncostatin M compared with the WT.

**Table 3:** Susceptibility of NS5A RAS to NS5B inhibitors and other reagents.

Cell line (OR6c/1B-4)	Sofosbuvir		Interferon-α		Fluvastatin		Oncostatin M	
	EC50 (nM)	Fold resistance	EC50 (ng/L)	Fold resistance	EC50 (µM)	Fold resistance	EC50 (ng/mL)	Fold resistance
WT	113.3	1.0	48.3	1.0	1.5	1.0	0.9	1.0
Q24K/L28M/R30Q/A92K	79.9	0.7	103	2.1	1.7	1.1	1.5	2.3
Q24K/L28M/R30E/A92K	100.5	0.9	96.2	2.0	2.7	1.8	3.5	3.9

The fold change in resistance was compared with that of the WT.

*EC50* effective concentration required to inhibit 50%, *WT* wild-type, *NS* nonstructural, *RAS* resistance-associated substitution.

### Effect of pibrentasvir and sofosbuvir on Q24K/L28M/R30E/A92K

**Figure 3:**
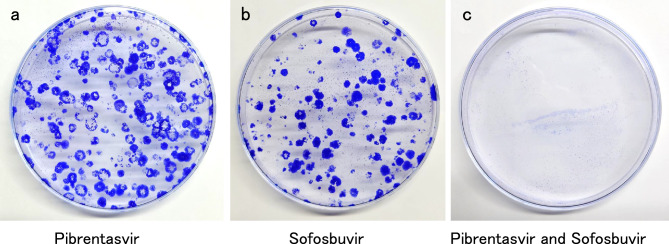
Effect evaluation of pibrentasvir and sofosbuvir on the NS5A Q24K/L28M/R30E/A92K by Coomassie Brilliant Blue staining. Ten-centimeter dishes were prepared in which the Q24K/L28M/R30E/A92K RASs were grown continuously, and each dish was supplemented with (**a**) pibrentasvir alone, (**b**) sofosbuvir alone, or (**c**) a combination of pibrentasvir and sofosbuvir.

The Q24K/L28M/R30E/A92K was more resistant to all NS5A inhibitors (specifically to pibrentasvir) than the WT and Q24K/L28M/R30Q/A92K. However, among the NS5A inhibitors examined in this study, pibrentasvir showed the weakest resistance to the Q24K/L28M/R30E/A92K RASs. Compared with WT, Q24K/L28M/R30E/A92K was not resistant to the NS5B inhibitor sofosbuvir. Therefore, we believe that the combination of pibrentasvir and sofosbuvir is most effective against Q24K/L28M/R30E/A92K, and we investigated the effect of this combination on the Q24K/L28M/R30E/A92K RASs (Fig. 3).

Ten-centimeter dishes were prepared in which the Q24K/L28M/R30E/A92K RASs grew continuously. Each dish was supplemented with either pibrentasvir alone (Fig. 3a), sofosbuvir alone (Fig. 3b), or a combination of pibrentasvir and sofosbuvir (Fig. 3c). Pibrentasvir and sofosbuvir were added at concentrations of 80 nM and 1 µM, respectively, which were 10 times the EC50 against the Q24K/L28M/R30E/A92K RASs. The agents were added each time the cells were passaged. G418 was not added for the first 2 weeks; subsequently, G418 was added at each passage to select the cells. After three passages, Coomassie Brilliant Blue (CBB) staining showed that the cells almost died in the dish containing both pibrentasvir and sofosbuvir, whereas cell proliferation continued in the dishes containing only pibrentasvir or sofosbuvir (Fig. 3). While single administration of pibrentasvir or sofosbuvir failed to suppress the proliferation of the Q24K/L28M/R30E/A92K RASs, the combination was able to suppress it. Therefore, it was considered that HCV with multiple mutations of NS5A Q24K/L28M/R30E/A92K could be treated effectively by the combined administration of pibrentasvir and sofosbuvir.

## Discussion

In the present report, we revealed the susceptibility of the coexistence of NS5A Q24K/L28M/R30E/A92K RASs in the NS5A region to various agents using subgenomic replicon cells. These RASs were highly resistant to all NS5A inhibitors in vitro*.* There have been no reports on the susceptibility of coexistence of these RASs to NS5A inhibitors in vitro. In addition, combination treatment with pibrentasvir and sofosbuvir against the coexistence of these RASs was effective in the replicon cells. Additionally, our study was performed using only HCV cells generated from genotype 1b, which may be clinically relevant.

In a nationwide multicenter study in Japan, a total of 1,193 patients with HCV for whom DAA treatment had failed were enrolled. NS5A L28M, R30Q, and A92K RASs were found in 17%, 17%, and 2.4% of patients, respectively^14^. Although this report did not mention the prevalence of the coexistence of Q24K/L28M/R30Q/A92K or Q24K/L28M/R30E/A92K RASs, it was speculated that cases with such mutations existed. Among the patients enrolled in the domestic phase III study of glecaprevir/pibrentasvir, there were some patients with RASs in NS5A Q24/L28/R30/A92 among those already treated with DAA. All of these patients achieved SVR with glecaprevir/pibrentasvir, except for those with the P32del mutation^15–17^. No patient had the coexistence of NS5A Q24K/L28M/R30E/A92K RASs. In actual clinical practice, our group and Sezaki et al. reported that the coexistence of NS5A Q24K/L28M/R30E/A92K RASs was observed in patients for whom glecaprevir/pibrentasvir treatment failed, and R30Q was changed to R30E after virologic failure^12,18^. However, the coexistence of Q24K/L28M/R30Q/A92K or Q24K/L28M/R30E/A92K RASs has appeared in re-treatment cases and has been reported in small numbers. We believe that to eradicate HCV globally and to prevent HCV-infected individuals from progressing to liver cirrhosis or developing cancer, establishing a treatment for highly resistant HCV is important.

According to the glecaprevir/pibretasvir interview form^17^, in an experiment using the Con-1 HCV strain, which is a replicon cell of genotype 1B, a single mutation of L28M or R30Q RASs was 1.0-fold or 0.5-fold resistant to pibrentasvir, respectively, compared with the WT. No resistance was observed with single mutations. Resistance to single mutations of Q24K, R30E, and A92K and resistance to multiple mutations of Q24K, L28M, R30Q, R30E, and A92K were not examined. Nitta et al. examined the NS5A region of the genotype 2a JFH-1 strain with the genotype 1b Con-1 HCV strain^19^. They generated strains with a single A92K mutation in the NS5A region and multiple RASs of NS5A Q24K, L28M, and R30Q, as reported in cases previously treated with daclatasvir and asunaprevir^16,20^. They examined these strains in cells transduced with Huh7.5.1 and showed that compared with the WT, the A92K RAS was 17,000,000-fold resistant to ledipasvir, 13.7-fold resistant to velpatasvir, and 8600-fold resistant to elbasvir. The R30Q/A92K RAS was 20,000,000-fold resistant to ledipasvir, 620-fold resistant to velpatasvir, and 31,000-fold resistant to elbasvir than the A92K single mutation, and the Q24K/L28M/R30Q/A92K RAS was more resistant than the R30Q/A92K RASs. However, susceptibility to the coexistence of NS5A Q24K/L28M/R30E/A92K RASs was not examined.

The mechanism by which HCV RASs occur remains unclear. Kai et al. revealed two mechanisms in post-treatment RASs: the selection of pre-existing substitutions among quasispecies and generation of novel mutations during therapy using deep sequencing analysis^21^. Yamauchi et al. suggested that the combination of various mutations, other than the known signature RASs, influences the kinetics of individual HCV quasispecies during DAA treatment^22^. They analyzed single‐molecule long‐read sequencing using rolling circle amplification to correct sequencing errors in the Oxford Nanopore sequencer. In the present study, we also performed ultra-deep sequencing analysis using samples taken before and after glecaprevir/pibrentasvir treatment. Case 1 had Q24K/L28M/R30Q/A92T RASs before daclatasvir and asunaprevir treatment, and novel mutations such as R30E and A92K RASs appeared after daclatasvir and asunaprevir treatment. The pre-existing R30E and A92K RASs before glecaprevir/pibrentasvir treatment induced glecaprevir/pibrentasvir failure. Case 2 also had R30E and A92K RASs before glecaprevir/pibrentasvir treatment. Most amino acid substitutions were Q24K/L28M/R30E/A92K RASs after glecaprevir/pibrentasvir treatment (Table 1). We speculate that the R30Q to R30E mutation we identified also occurred via the above two mechanisms.

Several studies have reported the effects of anti-HCV treatment on the subsequent course of patients. Laursen et al. showed that successful DAA therapy was beneficial in advanced liver disease, with an initial rapid resolution of liver inflammation and a subsequent gradual but steady improvement in liver fibrosis, metabolic liver function and reaction time^23^. Furthermore, Seko et al. reported that among patients with HCC who were treated with molecularly targeted agents, the overall survival of patients who achieved SVR was significantly longer than that of those who did not achieve SVR (18.1 months vs. 11.3 months)^24^. Ochi et al. reported that in Child-Pugh grade A patients with a history of hepatectomy or radiofrequency ablation for HCC, the DAA-treated group had a significantly higher survival rate at 48 months and a significantly lower HCC recurrence rate than the untreated group^25^. The SVR rate is generally > 95% owing to the progression of DAA.

Ikeda et al. reported mizoribine, fluvastatin, and teprenone as candidate anti-HCV agents^26–28^. Furthermore, they reported that oncostatin M, a member of the interleukin-6 family, had marked anti-HCV activity in an HCV RNA-replicating cell culture system and showed synergistic inhibitory activity against interferon-alpha even at low concentrations^29^. We wondered whether agents other than DAAs with anti-HCV activity could be added to DAAs to obtain an effect against the Q24K/L28M/R30E/A92K and investigated interferon-alpha, fluvastatin, and oncostatin M. The Q24K/L28M/R30Q/A92K and Q24K/L28M/R30E/A92K RASs were mildly resistant to these agents compared with the WT, and no additional treatment was considered (Table 3). The combination of pibrentasvir and sofosbuvir successfully eliminated HCV in the Q24K/L28M/R30E/A92K RASs (Fig. 3).

One possible limitation of this study is that replicon cells may have acquired new HCV mutations other than those at the insertion site during cell passaging, which may have affected the results. We sequenced 3 types of replicon cells after passing them 13 times to confirm that the cells had not acquired any new mutations. Furthermore, Kato et al. analyzed spontaneous genetic mutations in HCV replicon cells, 50-1 cells derived from HuH-7 cells, and sO cells after 9 years of passaging culture. The results showed that genetic mutations in both replicon cells accumulated at 2.3 × 10^3^ to 3.1 × 10^3^ base substitutions/site/year^30^. The cells used in this study were of the OR6c/1B-4 strain, which differs from those used in previous studies. However, only cells that had been passaged fewer than 20 times were used in the experiment, and the sustained passage period was as short as 2-3 months, suggesting that the acquisition of new mutations during the passage was quite low.

In Conclusion, in the subgenomic replicon cell analysis, the coexistence of NS5A Q24K/L28M/R30Q/A92K and Q24K/L28M/R30E/A92K RASs was highly resistant to daclatasvir, ledipasvir, velpatasvir, and elbasvir. The Q24K/L28M/R30Q/A92K RASs were mildly resistant to pibrentasvir, whereas the Q24K/L28M/R30E/A92K RASs were highly resistant to pibrentasvir. Pibrentasvir and sofosbuvir combination therapy may be effective against the coexistence of NS5A Q24K/L28M/R30E/A92K RASs. Combination regimens may eradicate HCV with NS5A Q24K/L28M/R30E/A92K RASs.

## Materials and methods

### Reagents

Pibrentasvir, velpatasvir, PSI-7977 (sofosbuvir), and fluvastatin were purchased from CAYMAN CHEMICAL (Ann Arbor, MI, USA), whereas daclatasvir, oncostatin M were purchased from R&D Systems (Minneapolis, MN, USA). Dimethyl sulfoxide and CBB R-250 were purchased from Sigma-Aldrich (St. Louis, MO). Dulbecco's modified Eagle’s medium and High Glucose (500 mL) were mixed with 5 mL of l-glutamine solution, 5 mL of modified Eagle’s medium non-essential amino acid solution, 5 mL of penicillin-streptomycin solution, and 50 mL of Fetal Bovine Serum before use. All reagents and phosphate-buffered saline (PBS) were purchased from Invitrogen (Waltham, MA, USA). The Antibiotic G-418 Sulfate Solution (G418) and *Renilla* Luciferase Assay System 1000 were purchased from Promega (Madison, WI, USA), 50% methanol from Fujifilm Wako Pure Chemicals Corporation (Osaka, JPN), and 10% acetic acid from Nacalai Tesque (Kyoto, JPN). KOD-201 was purchased from TOYOBO (Osaka, JPN).

### Cell cultures

OR6 cells were cloned from HuH-7 cells derived from ORN/C-5B, a genotype 1b HCV-O strain^27,31^. OR6c cells were healed OR6 cells in which HCV RNA was removed by interferon-alpha treatment^26^. OR6c cells were then electroporated with HCV 1B-4 replicon RNA with genotype 1b^32^, which contain *Renilla* luciferase genes and neomycin phosphotransferase. Subgenomic replicon cells were generated and designated as WT. The WT cells had HCV NS5A Q24Q/L28L/R30R/A92A. We also requested GENEWIZ (Tokyo, JPN) to synthesize artificial genes numbered 4660 to 7210, containing the coexistence of NS5A Q24K/L28M/R30E/A92K RASs. These genes were substituted into the NS5A region of the 1B-4 strains and electroporated to generate the Q24K/L28M/R30E/A92K RASs (Fig. 1). Furthermore, 1B-4 strains containing the artificial genes were subjected to a quick change from R30E to R30Q (from GAG to CAG) and electroporated into OR6c cells to generate the Q24K/L28M/R30Q/A92K RASs. The cells containing Q24K/L28M/R30E/A92K were incubated in a T100 Thermal Cycler (Bio-Rad) using primers containing R30Q, KOD-201 (KOD-Plus, 10 × Buffer for KOD-Plus, 25 mM dNTPs, 25 mM MgSO4), and dimethyl sulfoxide. The primers used for the quick changes were created by Hokkaido System Science Co., Ltd. Amino acids other than Q24/L28/R30/A92 were the same in all three RASs, with no additional mutations.

These cells contained the *Renilla* luciferase gene in HCV 1B-4 strains, allowing HCV RNA levels to be confirmed by measuring *Renilla* luciferase activity. Furthermore, these cells were designed to die under G418 administration when HCV RNA was removed or when HCV RNA levels decreased, as HCV 1B-4 strains contained neomycin phosphotransferase^27^ and were cultured in Dulbecco’s modified Eagle’s medium with G418. When WT, Q24K/L28M/R30Q/A92K, Q24K/L28M/R30E/A92K, and OR6c cells before electroporation with 1B-4 strains were cultured under G418 administration, only the OR6c cells died (Supplementary Figure). Colonies of established strains were selected, *Renilla* luciferase activity was measured, and the cells extracted from the colony with the highest activity were used in this study. Cells with fewer than 20 passages were used in this study.

### Renilla luciferase reporter assay

For the *Renilla* luciferase assay, 1.5 × 10^4^ cells/well were seeded in a 24-well plate; after 24 h of culture, anti-HCV agents were added, and the cells were treated for 72 h. Afterwards, the medium was removed, and the cells were washed with PBS and collected using 100 μL of *Renilla* luciferase assay lysis buffer. After shaking for 30 min, 50 μL of *Renilla* luciferase assay reagent (*Renilla* luciferase assay buffer and substrate were mixed 100:1) was added to 10 μL of collected cells. Finally, luciferase activity was measured using GloMaxⓇ20/20 Luminometer (Promega).

### CBB staining

Referring to previous studies^33,34^, 500 mL of 0.6% CBB was prepared by mixing 3 g of CBB R-250, 250 mL of 50% methanol, 50 mL of 10% acetic acid, and 200 mL of ultrapure water. The same composition (without CBB R-250) was used as the washing solution. After washing the 10 cm dish (to be stained) with PBS, 2 mL of 0.6% CBB was added, and the dish was stained for 5 min. The CBB was then removed, washed several times with 10 mL of the washing solution, and dried.

### Assessment of amino acid substitutions

We analyzed the amino acid substitutions in the NS5A regions of two patients with glecaprevir /pibrentasvir failure before and after treatment by direct sequencing and ultra-deep sequencing. We have previously reported in detail about the direct sequencing method^10,11^. An ultra-deep sequencing analysis was performed using the MiSeq® sequencing system (Illumina, San Diego, USA) with 150-bp paired-end reads, according to the manufacturer’s instructions as previously described in detail^35^. The results were mapped to the genome reference sequence in the CLC Genomics Workbench (CLC Bio, Aarhus, Denmark). HCV-Con1 (accession no. AJ238799) or HCV-J (accession no. D90208) were used as references. RASs in the NS5A region were evaluated for amino acid substitutions of Q24, L28, R30, L31, P32, F37, Q54, P58, A92, and Y93. Amino acid substitutions were defined when they were detected at a frequency greater than 0.2% of the total coverage.

### Statistical analysis

Statistical analysis was performed using two-way ANOVA with Dunnett’s multiple comparisons test. P-values less than 0.05 were considered statistically significant.

### Supplementary Information

Supplementary Figure 1.

## Data Availability

Data is provided within the manuscript.

## Abbreviations

HCV: Hepatitis C virus
NS: Nonstructural
DAAs: Direct-acting antivirals
SVR: Sustained virological response
RASs: Resistance-associated substitutions
CBB: Coomassie brilliant blue
PBS: Phosphate buffered saline
WT: Wild-type
EC50: Effective concentrations required to inhibit 50%
HCC: Hepatocellular carcinoma
RNA: Ribonucleic acid
