# Supplementary material for: Analysis of the susceptibility of refractory hepatitis C virus resistant to nonstructural 5A inhibitors
Source: Sci Rep. 2024 Jul 16;14:16363. doi: 10.1038/s41598-024-67169-5 (PMC11252252; doi:10.1038/s41598-024-67169-5)
Supplement: Supplementary file 1 — Supplementary Figure 1. [file 41598_2024_67169_MOESM1_ESM.pptx]

## Slide 1
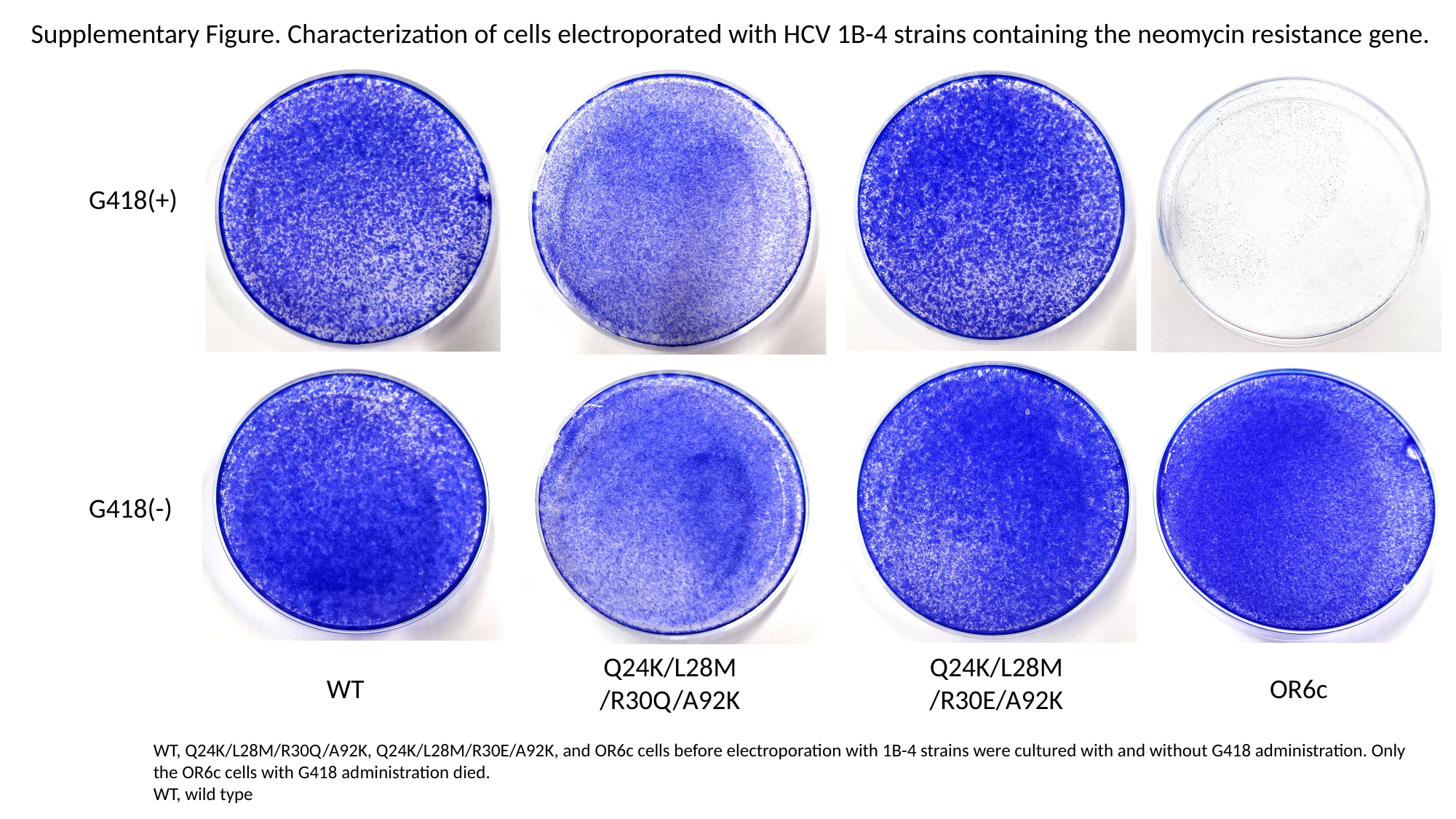

Supplementary Figure. Characterization of cells electroporated with HCV 1B-4 strains containing the neomycin resistance gene.
G418(+)
G418(-)
Q24K/L28M
/R30Q/A92K
Q24K/L28M
/R30E/A92K
WT
OR6c
WT, Q24K/L28M/R30Q/A92K, Q24K/L28M/R30E/A92K, and OR6c cells before electroporation with 1B-4 strains were cultured with and without G418 administration. Only the OR6c cells with G418 administration died.
WT, wild type
